# Hydrogen Peroxide-Resistant CotA and YjqC of *Bacillus altitudinis* Spores Are a Promising Biocatalyst for Catalyzing Reduction of Sinapic Acid and Sinapine in Rapeseed Meal

**DOI:** 10.1371/journal.pone.0158351

**Published:** 2016-06-30

**Authors:** Yanzhou Zhang, Xunhang Li, Zhikui Hao, Ruchun Xi, Yujie Cai, Xiangru Liao

**Affiliations:** 1 The Key Laboratory of Industrial Biotechnology, Ministry of Education, School of Biotechnology, Jiangnan University, 1800 Lihu Avenue, Wuxi, 214122, China; 2 The Bioscience and Engineering College, Jiangxi Agriculture University, Nanchang, 330045, China; 3 Institute of Applied Biotechnology, Taizhou Vocational & Technical College, Taizhou, 318000, China; 4 College of Forestry, South China Agricultural University, Guangdong Key Laboratory for Innovative Development and Utilization of Forest Plant Germplasm, Guangzhou, 510642, China; Loyola University Chicago, UNITED STATES

## Abstract

For the more efficient detoxification of phenolic compounds, a promising avenue would be to develop a multi-enzyme biocatalyst comprising peroxidase, laccase and other oxidases. However, the development of this multi-enzyme biocatalyst is limited by the vulnerability of fungal laccases and peroxidases to hydrogen peroxide (H_2_O_2_)-induced inactivation. Therefore, H_2_O_2_-resistant peroxidase and laccase should be exploited. In this study, H_2_O_2_-stable CotA and YjqC were isolated from the outer coat of *Bacillus altitudinis* SYBC hb4 spores. In addition to the thermal and alkali stability of catalytic activity, CotA also exhibited a much higher H_2_O_2_ tolerance than fungal laccases from *Trametes versicolor* and *Trametes trogii*. YjqC is a sporulation-related manganese (Mn) catalase with striking peroxidase activity for sinapic acid (SA) and sinapine (SNP). In contrast to the typical heme-containing peroxidases, the peroxidase activity of YjqC was also highly resistant to inhibition by H_2_O_2_ and heat. CotA could also catalyze the oxidation of SA and SNP. CotA had a much higher affinity for SA than *B*. *subtilis* CotA. CotA and YjqC rendered from *B*. *altitudinis* spores had promising laccase and peroxidase activities for SA and SNP. Specifically, the *B*. *altitudinis* spores could be regarded as a multi-enzyme biocatalyst composed of CotA and YjqC. The *B*. *altitudinis* spores were efficient for catalyzing the degradation of SA and SNP in rapeseed meal. Moreover, efficiency of the spore-catalyzed degradation of SA and SNP was greatly improved by the presence of 15 mM H_2_O_2_. This effect was largely attributed to synergistic biocatalysis of the H_2_O_2_-resistant CotA and YjqC toward SA and SNP.

## Introduction

Peroxidase and laccase have are two of the most promising oxidoreductase enzymes largely because they can catalyze the oxidation of a broad range of phenolic compounds [1]. They have been widely used for several applications, such as the degradation of aromatic pollutants, pulp delignification, lignin bioprocessing, bleaching, biosensors and biofuel cells [2, 3]. In these applications, most of the enzymatic processes were based on a single-enzyme catalysis of laccase or peroxidase. To better accommodate variable environments or diverse substrates, recent studies have revealed that a promising avenue would to develop a multi-enzyme biocatalyst comprising peroxidase, laccase and other enzymes (e.g., glucose oxidase) [4, 5]. Unfortunately, these studies did not examine the effects of hydrogen peroxide (H_2_O_2_) on laccase and peroxidase. Generally, peroxidases require H_2_O_2_ as a co-substrate to catalyze the oxidation of phenolic compounds, yet peroxidases are highly vulnerable to H_2_O_2_ [3]. Although laccase uses molecular oxygen rather than H_2_O_2_ as an electron acceptor, its activity can be reversibly inhibited by H_2_O_2_ under specific conditions. For example, H_2_O_2_ produced by glucose oxidase inhibited catalytic or bioelectrocatalytic performance of laccase in enzymatic fuel cells [6–8]. The inhibition of laccase or peroxidase by H_2_O_2_ has attracted more general interest in industrially relevant applications [3, 7]. Therefore, the H_2_O_2_-resistant peroxidase and laccase may be more useful for developing a multi-enzyme biocatalyst.

Recently, bacterial laccases have been recommended as a promising alternative for fungal laccases due to their high thermal stability and high alkaline tolerance [9]. The outstanding representives of bacterial laccases are thermo- and alkali-stable CotA laccases from *Bacillus subtilis* and other *Bacillus* species [2, 10, 11]. The CotA protein is located in the outer spore coat and contributes to protecting spores from UV light, H_2_O_2_ and other chemical agents. Thus, the CotA laccase is supposed to be more resistant to H_2_O_2_ or high salinity than fungal laccases [10, 12]. Compared with the extensive use of heat-, alkali- and halide-resistant CotA laccases, little attention has been focused on *Bacillus* CotA laccases with high resistance to H_2_O_2_ [12].

In addition to the protein CotA, there is evidence that the spore coat protein of sporulation-related manganese (Mn) catalase (YjqC) grants *B*. *pumilus* SAFR-032 spores a unique resistance to H_2_O_2_ [13]. However, the enzymatic propeties of YjqC have yet to be explored. Recent research suggests that Mn catalases of *Pyrobaculum calidifontis* and *Thermus thermophilus* display peroxidase activity toward phenolic compounds [14, 15]. Previously, the most applicable peroxidases mainly included fungal peroxidases and horseradish peroxidase. They both belong to a traditional group of heme-containing peroxidases that have inherent drawbacks, such as H_2_O_2_-induced inactivation and thermal instability. In contrast to traditional peroxidases, the peroxidase activity of catalases is considered to be more resistant against denaturation by heat and H_2_O_2_. These features make catalases an interesting alternative for typical peroxidase-based applications [16]. However, the full potential of catalases functioning as peroxidases remains unknown [14, 16, 17]. Thus, the peroxidase activity of YjqC toward phenolic compounds deserves further investigation.

As noted above, YjqC and CotA are both structural proteins of the spore outer coat, and they promote spore resistance to H_2_O_2_ [13, 18]. On the other hand, the spore coat layer may have potential to improve the H_2_O_2_ stability of YjqC and CotA. One argument for this possibility is that spore CotA is more thermostable and alkali-resistant than its free counterparts [19, 20]. Another piece of supporting evidence has is that enzymes immobilized onto the spore coat show enhanced enzyme stability against pH and temperature [21]. Recently, the spore outer coat has been used as excellent support surface for immobilization of enzymes [21, 22]. Thus, YjqC and CotA can be regarded as inherent enzymes immobilized on a spore surface [13, 23, 24]. Spores containing CotA have also been directly used as biocatalysts for degrading synthetic dyes [19, 20]. Spore biocatalysts can also contribute to preventing the purification and immobilization of enzymes [25]. Moreover, the production of spores is also simple and economically beneficial for industrial applications [22]. However, there has been no effort to date to explore spore enzymes, such as YjqC and CotA, as multi-enzyme biocatalysts. A multi-enzyme biocatalyst comprising YjqC and CotA may be more highly resistant to H_2_O_2_-induced inactivation that blocks the broader use of fungal laccases and peroxidases.

In this study, YjqC of *B*. *altitudinis* SYBC hb4 demonstrated peroxidase activity toward the phenolic compounds sinapic acid (SA) and sinapine (SNP, the choline ester of SA) with H_2_O_2_ as a co-substrate. CotA of *B*. *altitudinis* SYBC hb4, which is similar to homologous proteins from other *Bacillus* species, also exhibited catalytic oxidation toward SA and SNP [26–28]. Laccases and peroxidases typically transform phenolic substrates into oligomers that show less toxicity or lower bioactivity to organisms [5]. Apparently, CotA and YjqC of *B*. *altitudinis* SYBC hb4 may be two promising enzymes for the detoxification of SA and SNP. SA and SNP are two major antinutrients that hinder the development of valuable uses for rapeseed meal (RSM) [29]. The elimination of antinutrients is desirable for commercial utilization of RSM. It has been proposed that a multi-enzyme biocatalyst comprising different oxidative enzymes (e.g., laccase, peroxidases) are more preferable for the efficient degradation of phenolic compounds in biomass [4, 5, 30, 31]. Therefore, development of a synergistic multi-enzyme biocatalyst comprising CotA and YjqC may be necessary for efficient removal of SA and SNP from RSM. As mentioned above, spores just happen to have this multi-enzyme biocatalyst. In this study, *B*. *altitudinis* SYBC hb4 spores were assessed for biocatalysis toward SA and SNP. We also examined the possibility that *B*. *altitudinis* SYBC hb4 spores could be used for the degradation of SA and SNP in RSM.

## Materials and Methods

### Strain and Spore Production

The strain used for the experiments was *B*. *altitudinis* SYBC hb4 (*B*. *altitudinis* hb4), which was deposited in the China Center For Type Culture Collection (CCTCC No. M2015018). The strain was isolated from gall honey (the honeybee *Apis cerana* collected nectar from gall flowers), which was purchased from Apiary of Ba Ma Mi Yuan Co. Ltd., Bama, China [32, 33]. According to reports, an appropriate concentration of Mn^2+^ was often used for promoting *Bacillus* sporulation [34]. Therefore, 0.15 mM MnCl_2_ was used for improving production of spores by *B*. *altitudinis* hb4 in this study. Additionally, *Bacillus* CotA appears as a copper-dependent laccase [18, 35]. Thus, NMC medium (NB medium plus 0.15 mM MnCl_2_ and 0.25 mM CuSO_4_) was used for production of activated *B*. *altitudinis* hb4 CotA, whereas the NM medium (NB medium plus 0.15 mM MnCl_2_) was used for production of spores with small amounts of laccase activity. The NB medium was composed of 10.0 g L^-1^ peptone, 5.0 g L^-1^ beef extract, 5.0 g L^-1^ NaCl, pH 7.0–7.2 (with a background concentration of 0.3 μM Mn^2+^). The sporulation of *B*. *altitudinis* hb4 was initiated by inoculating 50 mL medium (loaded into 250-mL flask) with 1 mL of seed broth. The inoculated medium was incubated at 30°C with an oscillation speed of 200 rpm for 48 h. The chemicals and reagents were purchased from Sinopharm Chemical Reagent Co. Ltd., Shanghai, China.

### Enzyme Assay

Laccase activity was measured using spectrophotometry at 50°C with 2, 2'-azino-bis (3-ethylbenzothiazoline-6-sulfonic acid) (ABTS, Sigma-Aldrich Co. LLC., Shanghai, China) as a substrate [28]. The oxidation of ABTS was monitored at 420 nm (ε_420_ = 36 mM^-1^ cm^-1^). One unit of laccase activity (U) was defined as the amount of laccase required to oxidize 1 μmol ABTS per minute. Catalase activity was determined at 30°C by monitoring the decreased absorbance value of H_2_O_2_ (Aladdin Chemical Reagent Co. Ltd., China) at 240 nm (ε_240_ = 43.6 M^-1^ cm^-1^) [33]. One unit of catalase activity (U) was defined as the amount of catalase required to decompose 1 μmol H_2_O_2_ per minute. Assays were conducted in a reaction mixture consisting of 2.4 mL 0.1 M citrate-phosphate (Sinopharm, China) buffer, 0.1 mL spore suspension or free enzyme, and 0.5 mL 1 mM ABTS or 50 mM H_2_O_2_. The activities of the spores and free enzymes were expressed in terms of U OD_580_ of spores^-1^ and U mg^-1^ protein, respectively. In addition, isoenzyme analyses of catalases and laccases were performed with 8% polyacrylamide gel (Sinopharm, China) electrphorsis (PAGE) under non-denaturing conditions. The activity staining for catalases was conducted using a chromogenic probe containing isoniazid and pyrocatechol (Aladdin, China) [33]. The laccases were visualized with 10 mM DMP (Sigma, China) [36].

### Harvest and Purification of Spores

The harvest and purification of spores were performed as previously described [13, 23]. Briefly, the spores were harvested by centrifugation (8000 rpm, 20 min, 4°C), and then the pellets were suspended in wash solution containing 2.5 μg mL^-1^ MgSO_4_, 200 μg mL^-1^ lysozyme and 2 μg mL^-1^ DNase I (all purchased from Sinopharm, China). After 30 min of incubation at 37°C, the suspensions were heat-treated (80°C, 45 min) to ensure destruction of residual vegetative cells. The purified spores were free (>99%) of growing cells, germinated spores and cell debris. The purification was checked using a light microscope (Nikon Eclipse E200MV, Japan) and transmission electron microscopy (Hitachi TEM system, Japan). The purified spores were re-suspended in 0.1 M citrate-phosphate buffer (pH 7.0) at −20°C until use.

### Isolation of Spore Coat Proteins

The spores were extensively washed with distilled water and then decoated with the alkaline decoating method [37]. Briefly, the spore coat layer was stripped with a detergent solution consisting of 0.1 M NaOH, 0.1 M NaCl, 0.1 M dithiothreitol (DTT) and 0.5% sodium dodecyl sulfate (SDS). The stripping process involved incubating spores in 1 mL of the detergent solution at 70°C for 30 min. Then the residual spores were removed by centrifugation, and the supernatant was dialyzed against deionized water for 24 h in an ice bath. After dialysis, the supernatant was concentrated using an ultrafiltration centrifuge tube (Millipore, Bedford, MA). A blend of the concentrated supernatant and loading buffer (62.5 mM Tris-HCl, 10% glycerol, 2% SDS, 1% 2-mercaptoethanol, 0.003% bromophenol blue, pH 6.8) was boiled for 5 min. The boiled solution (approximately 50 μL) was analyzed with 12% SDS-PAGE and stained with Coomassie brilliant blue R-250. The clear bands were cut horizontally into slices (1.5 mm wide) for MALDI-TOF MS analysis. Dithiothreitol and 2-mercaptoethanol were both purchased from Sigma-Aldrich (Shanghai, China), and the rest of the ingredients were purchased from Sinopharm (Shanghai, China).

### MALDI-TOF MS Analyses of Spore Coat Proteins

The MALDI-TOF MS analyses of proteins were performed as described earlier [33]. In-gel digestion of proteins was conducted using the modified method described previously [13]. For identifying proteins, Mascot 2.1 (Matrix Science, http://www.matrixscience.com) was utilized to analyze mass spectrometry data. The parameters used by Mascot were as follows: trypsin was the specific enzyme; the ions score was -10_*_Log (P), where P was the probability that the observed match was a random event; individual ions scores > 57 indicated identity or extensive homology (*P* < 0.05); and protein scores were derived from ions scores as a non-probabilistic basis for ranking protein hits.

### Purification of CotA and YjqC from Spores

The spores were suspended in 0.1 M Tris-HCl buffer (pH 7.6) containing 1 mg mL^-1^ lysozyme and 1 mM phenylmethanesulfonyl fluoride (protease inhibitor, Sinopharm), then incubated at 37°C for 1 h with constant oscillation at 120 rpm. Then the spores were subjected to an intermittent ultrasonic crush for 30 min on ice. The crude lysate was separated from cellular debris by centrifugation at 10,000 rpm at 4°C for 30 min. The resulting supernatant was the crude enzyme. The purification of CotA and YjqC was conducted using a previously described method [33]. The main steps of the purification were, in order, fractional precipitation, ion exchange and gel filtration chromatography. The main procedures were performed on an Äkta Avant system (GE-Healthcare). The protein concentration of each fraction was determined with a Bradford assay. The purity of the protein fractions was analyzed by 12% SDS-PAGE.

### Characterization of CotA and YjqC

To characterize CotA and YjqC, the effects of temperature, pH and substrate concentration on their activity were assayed. The effects of pH and temperature on the activity of CotA toward ABTS were determined for pH values ranging from 3.0 to 8.0 (50 mM citrate-phosphate buffer) and temperatures ranging from 25 to 90°C. The stability of CotA under the optimum pH and temperature was determined by monitoring residual laccase activity during a 12 h incubation in 50 mM citrate-phosphate buffer. The optimal kinetic parameters of CotA were determined using different concentrations of ABTS (0.05–0.35 mM). The Michaelis-Menten constant (*K*_*M*_) and maximal reaction velocity (*Vmax*) of CotA were estimated by linear regression from double reciprocal plots according to a Lineweaver and Burk plot. The assays were also used for characterizing YjqC. In the assays, the laccase and catalase activity were both defined as relative activity, and the highest activity was regarded as 100%. Spore YjqC and spore CotA were also characterized with these assays but the free enzyme was replaced with spore suspension.

### CotA- and YjqC-Catalyzed Oxidation of SA and SNP

The CotA- and YjqC-catalyzed oxidation of SA and SNP were measured using a previously described method [28]. Briefly, the assay was performed in a reaction mixture containing 2.4 mL 50 mM citrate–phosphate buffer, 0.5 mL substrate (SA or SNP), and 0.1 mL of the enzyme solution or spore suspension. The oxidation of SA and SNP were monitored by decreases in absorbance at 307 nm (ε_307_ = 16,500 M^-1^ cm^-1^) and 326 nm (21,350 M^-1^ cm^-1^), respectively [38]. An appropriate concentration of H_2_O_2_ was required for initiating the peroxidase activity of YjqC toward SA and SNP. For the YjqC-catalyzed oxidation of SA and SNP, a co-substrate of 0.1 mL 50 mM H_2_O_2_ was added to the reaction mixture to drive the reaction. Meanwhile, the same methods used for CotA were used to determine the optimal YjqC-catalyzed oxidation of SA and SNP. The kinetic parameters of CotA and YjqC toward SA and SNP were also assayed. The effects of H_2_O_2_ concentrations (0–30 mM) on the peroxidase activity of YjqC were also assayed. SA and SNP were both purchased from Baoman Biotech Co. Ltd., Shanghai, China.

### H_2_O_2_ Stability Assay

The H_2_O_2_ stability of YjqC and CotA was determined by a method described earlier [3]. The half-inhibition concentration (IC_50_) values of H_2_O_2_ against the activity of CotA were determined by incubating free CotA with variable H_2_O_2_ concentrations (0–40 mM). The half-life of CotA was determined by incubating CotA with different concentrations of H_2_O_2_ (0–30 mM) for 0–180 min. The time (min) until the enzymes exhibit 50% residual activity was used to compare the activity stability of CotA under different concentrations of H_2_O_2_. The concentration of H_2_O_2_ that inhibited 50% of CotA residual activity was used for determining the tolerance of CotA to inhibition by H_2_O_2_. The residual activity was determined following the same procedure as the activity assay for CotA.

### RSM Treated with Spores

Samples of RSM were purchased from the local market in Wuxi, China. The spore suspension was prepared by suspending spores in 0.1 M citrate-phosphate buffer. Approximately 10 g of RSM was blended with 40 mL of treatment agent in a 50-ml capped test tube and incubated at 45°C for 18 h. The treatment agents were prepared as the spore suspension (OD_580_ = 10) plus 0 or 15 mM H_2_O_2_. RSM treated with the isometric citrate-phosphate buffer was used as a control. During 18 h of incubation, residual RSM was collected every 6 h for measuring SA and SNP.

### Extraction and Quantification of SA and SNP

SA and SNP were extracted from RSM using a modified method from Niu et al [39]. Briefly, extraction was conducted using refluxing with 80% ethanol for 4.5 h at 80°C (the solvent-to-RSM ratio was 15:1 v/w). The extracted solutions were collected and concentrated with a rotary evaporator. The concentrated solution was analyzed for SA and SNP using HPLC.

The quantification of SA and SNP was performed using Eclipse XDB-C18 column (Agilent, 5 μm, 4.6×250 mm) by Chromaster CM5110 with a DAD detector (HITACHI, Japan) following a previously described method [40]. Briefly, gradient elution was conducted using 10% methanol containing 2% acetic acid (pH 3.2) as solvent A, and 100% methanol as solvent B. The gradient of solvent B was set as 10, 20, 45, 70, 100, 100 and 10% B at 0, 7, 20, 25, 28, 31 and 40 min, respectively. The column was incubated at 25°C with a 0.8 mL/min flow rate. A volume of 10 μL of each sample was injected into the HPLC-DAD for analysis via an automatic sampler (Hitachi CM 5210 Auto Sampler). The chromatograms were acquired at 330 nm. The relative retention times of SA and SNP were identified by comparing peaks with their authentic standards. The contents of SA and SNP were quantified using the calibration curves generated from plotting the concentration of their corresponding standards against the area. The contents of SA and SNP in RSM were both expressed as the amount (mg) of SA or SNP per gram of RSM (mg/g).

### Statistical Analysis

The experiments were repeated 3 times with 3 replicates for the spore prepartions, RSM treatments, and extractions of SA and SNP. The assays were performed in triplicate for characterizing CotA and YjqC, as well as the catalytic oxdation of SA and SNP. The results were expressed as the mean values and standard deviations (Mean ± SD). Data was statistically evaluated with SPSS base 16.0 software. All the figures were drawn using Origin 8.0.

## Results and Discussion

### Localization of CotA and YjqC in the Spore Coat

**Fig 1:**
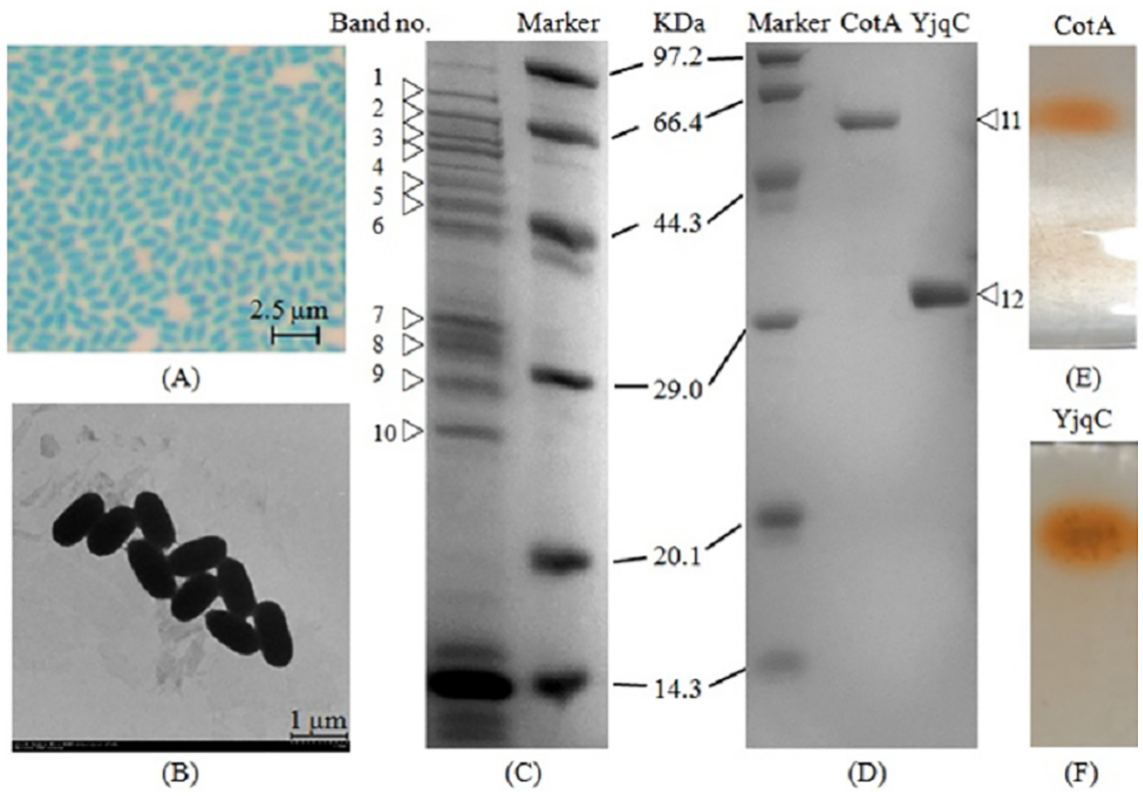
(A) Light micrograph of spores stained with malachite green. (B) Transmission electron micrograph of spores. (C) SDS-PAGE of the spore coat fraction extracted from *B*. *altitudinis* SYBC hb4 spores. (D) SDS-PAGE of the purified CotA and YjqC. (E) PAGE of laccases extracted from spores. (F) PAGE of catalases extracted from spores.

As shown in Fig 1A and 1B, the purified spores were free of vegetative cells and cellular debris. The average size of the spores was approximately 1.2 μm in length and 0.5 μm in width. The spore coat extracts were seperated and analyzed by 12% SDS-PAGE (Fig 1C). Ten distinct protein bands were excised and analyzed by MALDI-TOF MS (Band no. 1–10). The results (summarized in Table 1) indicated that the fourth and seventh bands were identified as CotA and a sporulation-related manganese catalase (YjqC), respectively. Evidently, the spore coat extracts of *B*. *altitudinis* hb4 were similar to extracts of other *Bacillus* species (e.g., *B*. *subtilis* and *B*. *pumilus*). SOD was also detected in the spore coat extracts (the tenth band) of *B*. *altitudinis* hb4. SOD was shown to be an indispensable protein involved in the maturation of spores [13].

**Table 1:** Identification of proteins extracted from spore coat fraction of *B*. *altitudinis* SYBC hb4.

BandNo.^a^	Identified protein	Accession No.	Mass	%Sequencecoverage	Mascotscore ^b^	Peptide (s)
1	Endopeptidase	WP_012011662.1	86,455	2	88	R.VLGPGGSGTTENVLAGIDR.A
2	Peptide ABC	WP_007496735.1	66,300	8	240	K.VAEVIQSNFEK.V
	transporter ATP					K.FHDGTDFNAEAVK.F
	binding protein					K.SGDSFRENPVGTGPFK.F
3	Oligopeptide	WP_007499033.1	61,986	6	191	K.VREDIAVVVQQQLK.E
	binding protein					K.HILGDVPIKDLGENEFNR.K
4	CotA	WP_012009087.1	58,882	6	117	R.DFEATGPFFER.E
						K.VWPYLEVEPR.K
5	Spore peptidoglycan	WP_007496056.1	50,407	8	96	K.AVSPQQAIAIAR.E
	hydrolase					R.ENNVSILYDETAQAPYFR.Y
6	Hypothetical protein	WP_012011041.1	49,057	3	76	R.LSQGEFLFTFDAR.D
7	Sporulation related	WP_012010687.1	31,115	21	254	R.ANLNAESQGR.L
	manganese catalase					R.EGDVVVPTTFPR.S
	(YjqC)					K.QQVSYDLFNFSR.G
						K.ELEEREGDVVVPTTFPR.S
8	Spore coat-associated	WP_007501219.1	29,157	7	97	K.ENSGAINLANIKPGDR.I
	protein					
9	Transglutaminase	WP_012011070.1	28,507	10	99	K.SGASFAIFQR.S
						R.QASDLLFEVTLR.S
10	SOD	WP_007501264.1	22,397	24	233	K.HHNTYVTNLNK.A
						R.FGSGWAWLVVNNGK.L
						K.LEITSTPNQDSPLTEGK.T
11	Purified CotA	WP_012009087.1	58,882	10	138	R.DFEATGPFFER.E
						K.VWPYLEVEPR.K
						R.TLTLTGTQDK.Y
						R.LGSVEVWSIVNPTR.G
12	Purified YjqC	WP_012010687.1	31,115	33	427	K.ELQYEAKPSKPDPLYAK.K
						K.YIIASGNLMADFR.A
						R.ANLNAESQGR.L
						R.EGDVVVPTTFPR.S
						K.QQVSYDLFNFSR.G
						K.ELEEREGDVVVPTTFPR.S

^*a*^ Band No. of proteins marked in Fig 1C and 1D

^*b*^ Individual ions scores > 57 indicate identity or extensive homology (*P* < 0.05)

Previously, more than 91 spore coat proteins have been identified in *B*. *subtilis* via SDS-PAGE, 2-DE gels, analysis of protein localization, genome-wide transcriptome and ‘‘gel-free” protocols [23]. In this study, only ten proteins were identified in the spore coat fraction, which likely occurred due to the sole use of SDS-PAGE for protein separation. The spore coat extraction method might also need improvement. Currently, YjqC has been detected in *B*. *subtilis* and *B*. *pumilus* according to the latest information from the NCBI database (http://www.ncbi.nlm.nih.gov/gene/). Based on the above MALDI-TOF MS analyses of spore coat proteins, *B*. *altitudinis* hb4 contains a homologous protein of *B*. *pumilus* YjqC.

In our previous work, the *yjqC* gene (KF857273) was cloned from the genome of *B*. *altitudinis* hb4. The deduced amino acid sequence (AHI58963.1) was obtained for phylogenetic analysis and conversation analysis. The sequence conservation (Table 2) showed that *B*. *altitudinis* hb4 YjqC had a 99% sequence identity to homologous proteins in *B*. *altitudinis*, *B*. *pumilus* and *B*. *aerophilus*. In contrast, the sequence exhibited 67, 84 and 84% sequence identity to homologous proteins from *B*. *licheniformis*, *B*. *subtilis* and *B*. *amyloliquefaciens*, respectively. The phylogenetic tree also indicated that YjqC of *B*. *altitudinis* hb4 had the closest relationship to *Bacillus pumilus* manganese catalase (Fig A in S1 File). Moreover, the peptides of homologous proteins identified by MALDI-TOF MS could perfectly match with the deduecd amino acid sequence of *B*. *altitudinis* hb4 YjqC. Thus, *B*. *altitudinis* hb4 YjqC could be identified as a Mn catalase.

**Table 2:** Sequence conservation (shown as % identity) of CotA and YjqC among *Bacillus* species.

Protein	% identity of *B*. *altitudinis* SYBC hb4 VS.					
	*B*. *altitudinis*	*B*. *pumilus*	*B*. *aerophilus*	*B*. *subtilis*	*B*. *amyloliquefaciens*	*B*. *licheniformis*
CotA	99	99	99	68	66	61
YjqC	99	99	99	84	84	67

Similar outcomes were also observed for CotA in *B*. *altitudinis* hb4. The cloning procedure for the *B*. *altitudinis* hb4 CotA gene (KU363621) was similar to that of *yjqC* [33]. The deduced amino acid sequence showed high sequence homology (99%) to CotA of *B*. *altitudinis*, *B*. *pumilus* and *B*. *aerophilus* (Table 2). In contrast, the homologous CotA from *B*. *licheniformis*, *B*. *amyloliquefaciens* and *B*. *subtilis* exhibited 61, 66 and 68% sequence identity with *B*. *altitudinis* hb4 CotA, respectively. The phylogenetic tree also suggested that *B*. *altitudinis* hb4 CotA shared a close relationship with CotA of *B*. *altitudinis* and *B*. *pumilus* (Fig A in S1 File).

From the above results, *B*. *altitudinis* hb4 CotA and YjqC both have extensive homology to homologous proteins of *B*. *pumilus*. *B*. *pumilus* exhibited a higher resistance against H_2_O_2_ and heat than other Bacilli, such as *B*. *subtilis* or *B*. *licheniformis* [41]. Recently, a heat stable CotA laccase has been identified in *B*. *pumilus* [42]. According to polyphasic taxonomy, the *B*. *pumilus* group comprises five species, including *B*. *pumilus*, *B*. *safensis*, *B*. *altitudinis*, *B*. *xiamenensis* and *B*. *invictae* [43]. Therefore, we could propose that *B*. *altitudinis* might exhibit similar H_2_O_2_ resistance to that of *B*. *pumilus*. Our previous work also strongly suggested that *B*. *altitudinis* hb4 was highly resistant to H_2_O_2_ [32, 33]. The enhanced resistance of *B*. *pumilus* spores to H_2_O_2_ was largely attributed to the spore coat proteins CotA and YjqC [13, 41]. However, past studies paid little attention to the H_2_O_2_ resistance of CotA and YjqC in *B*. *pumilus* or *B*. *altitudinis*. The research presented in this study demonstrated that *B*. *altitudinis* hb4 CotA and YjqC were highly resistant to H_2_O_2_.

### Purification of YjqC and CotA

The zymogram analyses showed that *B*. *altitudinis* hb4 spores harbored only one laccase and catalase (Fig 1E and 1F). The YjqC and CotA were purified from *B*. *altitudinis* hb4 spores using the multistep processes listed in Table 3. Analysis by SDS-PAGE (Fig 1D) showed that the purified catalase and laccase appeared as two single bands at ~31 KDa (Band no. 12) and ~58 KDa (Band no. 11), respectively.

**Table 3:** Purification of YjqC and CotA from *B*. *altitudinis* SYBC hb4 spores.

Purification step	Total activity (U)		Total protein (mg)		Specific activity (U mg^-1^)		Yield (%)		Purification (fold)	
	CotA	YjqC	CotA	YjqC	CotA	YjqC	CotA	YjqC	CotA	YjqC
Crude extract	30.4	46240	121	121	0.25	382.1	100	100	1	1
40–80% (NH_4_)_2_ SO_4_	23.3	25110	48.6	36.4	0.48	689.8	76.6	54.3	1.9	1.8
DEAE sepharose FF	17.0	7985	13.5	8.9	1.26	897.2	55.9	17.3	5.0	2.3
Superdex 200	9.6	5691	5.3	1.8	1.81	3161.7	31.6	12.3	7.2	8.3

**Fig 2:**
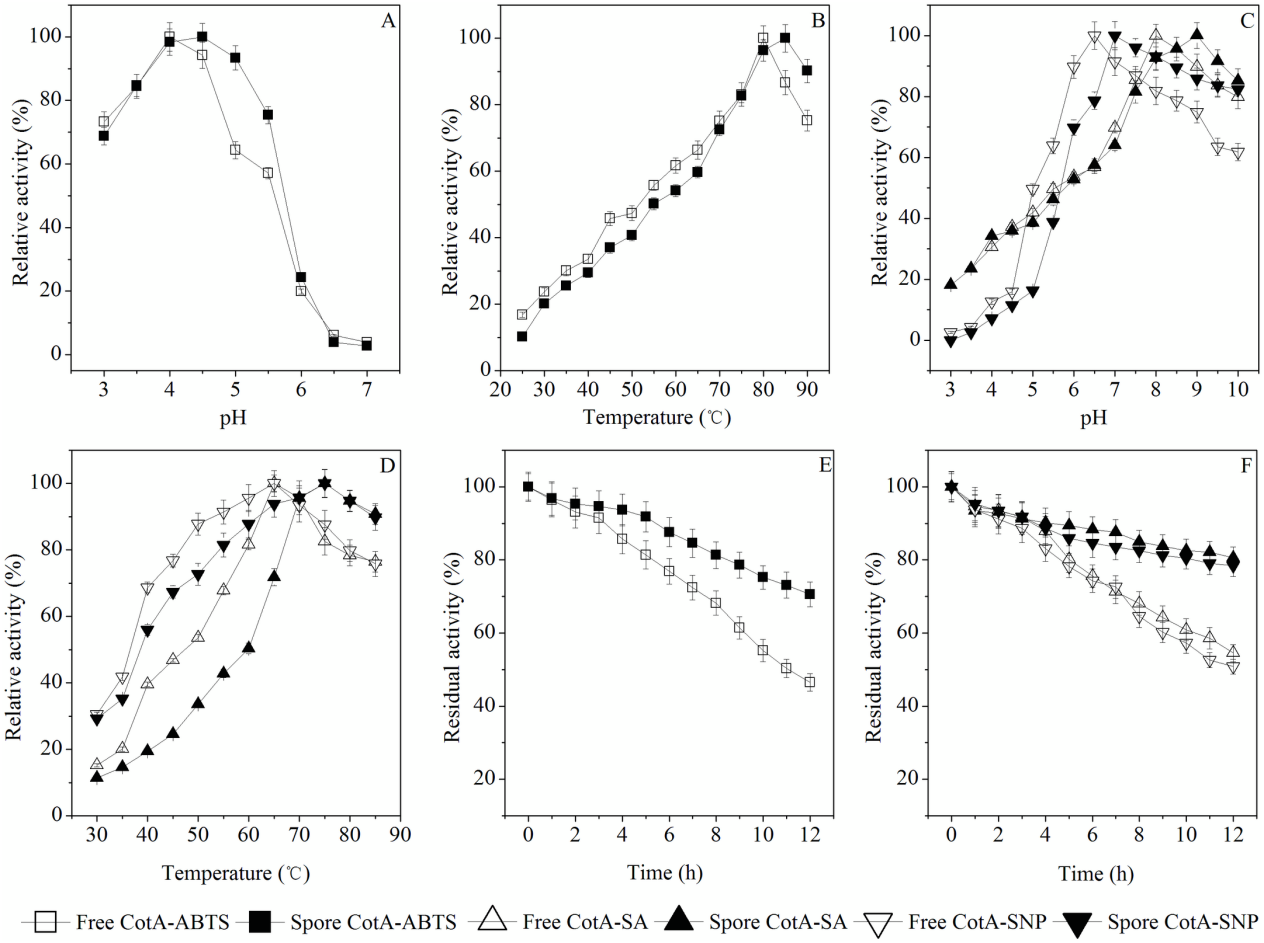
Optimal activity of the free and spore CotA. Laccase activity toward ABTS under different pH (A) and temperature (B) conditions. Laccase activity toward SA and SNP under different pH (C) and temperature (D) conditions. Stability of laccase activity toward ABTS (E), SA and SNP (F) under optimal pH and temperature conditions.

**Fig 3:**
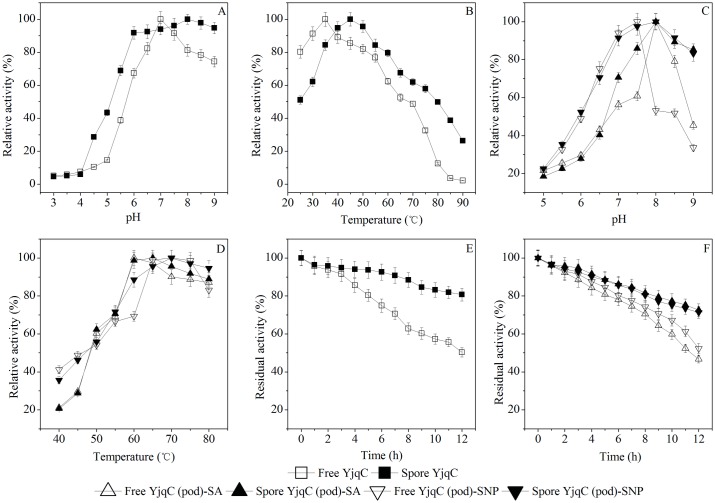
Optimal activity of the free and spore YjqC. Catalase activity under different pH (A) and temperature (B) conditions. Peroxidase activity toward SA and SNP under different pH (C) and temperature (D) conditions. Stability of catalase (E) and peroxidase (F) activity under optimal pH and temperature conditions.

**Fig 4:**
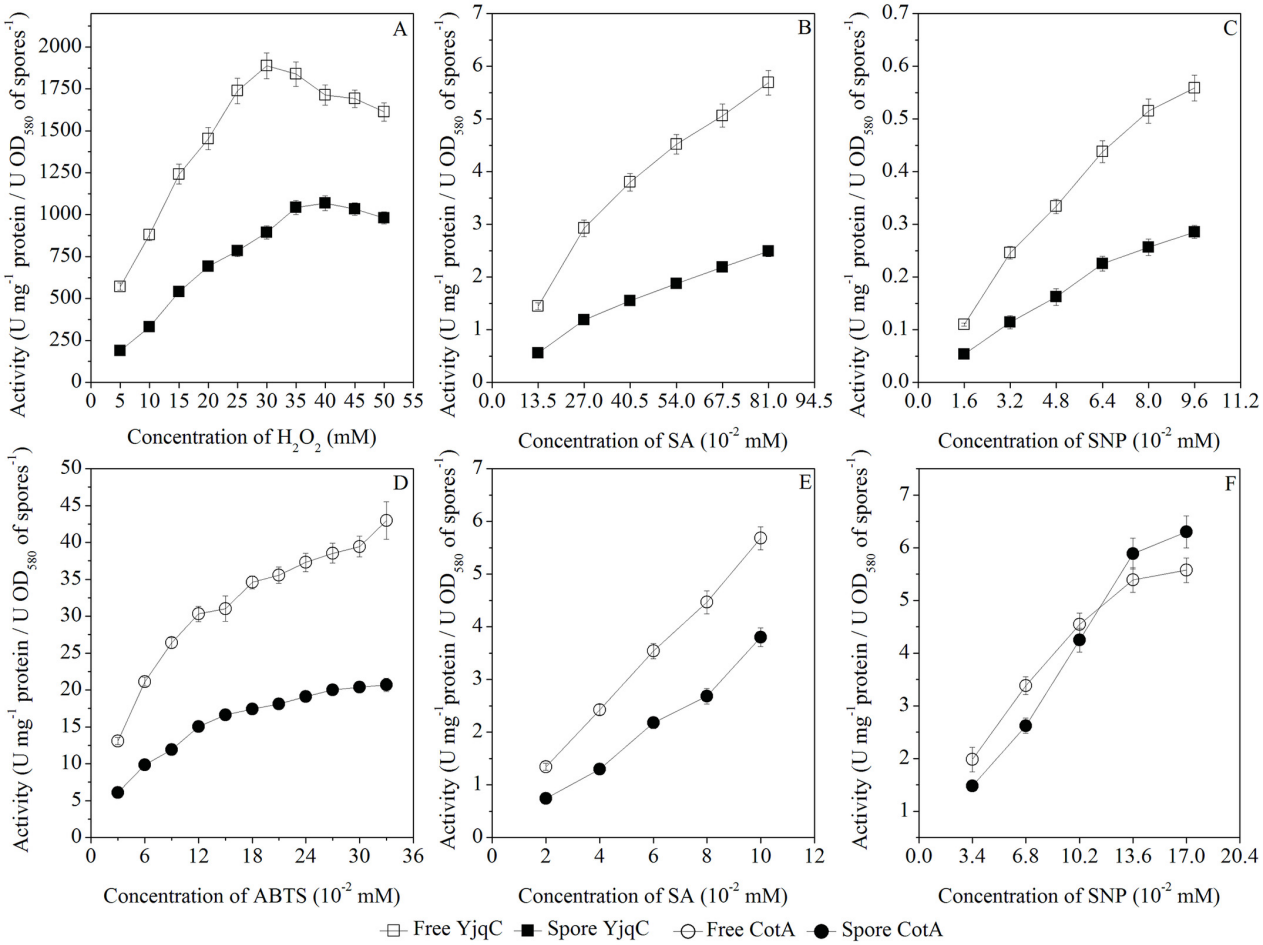
Effects of substrate concentrations on enzyme activity. Catalase activity of the free and spore YjqC under different concentrations of H_2_O_2_ (A). Peroxidase activity of the free and spore YjqC toward different concentrations of SA (B) and SNP (C). Laccase activity of the free and spore CotA toward different concentrations of ABTS (D), SA (E) and SNP (F).

These two bands were also analyzed by the MALDI-TOF MS. The identified peptides of the purified catalase and laccase were listed in Table 1. The results showed that the purified catalase and laccase were homologous YjqC and CotA from *B*. *pumilus*. Moreover, these identified peptides could match fragments of the deduced amino acid sequences for *B*. *altitudinis* hb4 YjqC and CotA. The YjqC was purified 8.3-fold with a final yield of 12.3%. The purified YjqC showed approximately 3161.7 U mg^-1^ protein of specific activity. The specific activity of the purified CotA was approximately 1.81 U mg^-1^ protein (with ABTS as a substrate). The CotA was purified 7.2-fold with a final yield of 31.6%. In the following, catalytic activity and stability of the purified CotA (Fig 2) and YjqC (Fig 3) toward substrates were determined under different pH and tempertures conditions. Meanwhile, the purified CotA and YjqC were also assayed for kinetic paramater under different concentrations of substrates (Fig 4).

### Characterization of CotA

As shown in Fig 2A and 2B, the free CotA could catalyze oxidization of ABTS for a pH range of 3.0–7.0 and a temperature range of 25–90°C. CotA showed optimum activity toward ABTS at pH 4.0 and 80°C. This outcome was similar to the optimal conditions for laccases from other *Bacillus* species (Table A in S2 File). The free CotA showed gradually increased laccase activity under 0.03–0.33 mM ABTS (Fig 4D). The dependence of the oxidation rate on ABTS concentration followed Michaelis–Menten kinetics. The kinetic parameters of CotA were determined and summarized in Table 4.

**Table 4:** Characteristics of YjqC and CotA purified from *B*. *altitudinis* SYBC hb4 spores.

Enzyme	Substrate	*K*_M_(μM)	*Vmax* (U mg^-1^ or OD_580nm_^-1^)	*k*_cat_ (s^-1^)	*k*_cat_*/K*_M_ (μM^-1^s^-1^)	*V*_max_*/K*_M_ ^b^
Free YjqC/Spore YjqC	H_2_O_2_	23525/102815	3195/4010	1650/-	0.07/-	-/0.039
	H_2_O_2_+SA^a^	1297/2295	15.62/10.17	8.1/-	0.006/-	-/0.004
	H_2_O_2_+SNP^a^	1600/8000	11.32/27.01	5.8/-	0.004/-	-/0.003
Free CotA/Spore CotA	ABTS	88/107	51.45/27.6	50.3/-	0.57/-	-/0.26
	SA	308/909	21.79/33.8	21.3/-	0.069/-	-/0.037
	SNP	163/1077	11.54/47.6	11.3/-	0.069/-	-/0.044

^*a*^ The concentration of H_2_O_2_ was 20 mM

^*b*^ The unit was expressed as min^-1^ OD_580nm_ of Spores^-1^

The *K*_M_ value of the free CotA toward ABTS was 88 μM. The free CotA had a higher affinity for ABTS than CotA of *B*. *subtilis* and laccase-like multicopper oxidase of *B*. *clausii* (Table A in S2 File). Its *K*_M_ value was much higher than the value for laccase from *B*. *licheniformis*, *B*. *coagulans* and *B*. *vallismortis*. The free CotA of *B*. *altitudinis* hb4 showed a similar affinity for ABTS to *B*. *pumilus* CotA. However, its *k*_cat_/*K*_M_ was lower than the value for *B*. *pumilus* CotA. In conclusion, compared with laccases from *Bacillus* species, *B*. *altitudinis* hb4 CotA did not appear to be a powerful catalyst for the oxidation of ABTS.

### Characterization of YjqC

The free YjqC was active within a pH range of 3.0–9.0 with optimal activity at pH 7.0 (Fig 3A). It was active in the temperature range of 25–90°C with a maximum activity at 35°C (Fig 3B). Its catalase activity showed a trend of rising first and then falling when exposed to 5–50 mM H_2_O_2_ (Fig 4A). The catalase activity of YjqC followed Michaelis–Menten kinetics when the concentration of H_2_O_2_ was less than 30 mM. The kinetic parameters of YjqC are also summarized in Table 4. The free YjqC exhibited a *K*_M_ of 23.5 mM and a *k*_cat_*/K*_M_ of 0.07 μM^-1^s^-1^. Compared to typical Mn catalases from *L*. *plantarum* and *T*. *thermophilum* (Table B in S2 File), *B*. *altitudinis* hb4 YjqC showed a much higher affinity toward H_2_O_2_. However, the *k*_cat_*/K*_M_ value of YjqC was lower than the value of Mn catalases from *P*. *calidifontis*, *L*. *plantarum*, *T*. *thermophilum*, *T*. *album* and *T*. *fusca* (Table B in S2 File). Although these enzymes are widely distributed in *Bacillus* spores, there are unfortunately few reports about the kinetic properties of *Bacillus* Mn catalases.

### CotA-Catalyzed Oxidation of SA and SNP

As shown in Fig 2C and 2D, the free CotA could catalyze oxidization of SA and SNP in a pH range of 3.0–10.0 and a temperature range of 30–85°C. The optimal conditions for CotA to oxidize SA were pH 8.0 and 65°C. The optimal conditions for CotA to oxidize SNP were pH 6.5 and 65°C. With increases in SA (0.02–0.10 mM) and SNP (0.034–0.17 mM), the free CotA exhibited increased catalytic activity (Fig 4E and 4F). The kinetic parameters of the free CotA toward SA and SNP were determined and summarized in Table 4. The *K*_M_ value of the free CotA was approximately 308 μM for SA. Compared with fungal laccases, *B*. *altitudinis* hb4 CotA exhibited a lower affinity toward SA (Table C in S2 File), but it had much higher affinity for SA than *B*. *subtilis* CotA. The catalytic efficiency of *B*. *altitudinis* hb4 CotA was also lower than fungal laccases and *B*. *subtilis* CotA. Previous studies have demonstrated that *Bacillus* CotA is a potent biocatalyst used for bioconversion of SA into valuable derivatives [44]. The kinetic parameters of *B*. *altitudinis* hb4 CotA were comparable to other CotA from *B*. *subtilis* and *B*. *licheniformis* [27, 28]. However, the CotA-catalyzed oxidation of SNP received less attention. The above results suggest that CotA is also efficient in catalyzing the oxidation of SNP. The free CotA showed similar *k*_cat_/*K*_M_ values (both approximately 0.069 μM^-1^ s^-1^) toward SA and SNP. However, from the *K*_M_ values, the free CotA showed a much higher affinity toward SNP than toward SA. According to recent studies, the kinetic parameters of CotA toward SNP have been reported only a few times.

### YjqC-Catalyzed Oxidation of SA and SNP

**Fig 5:**
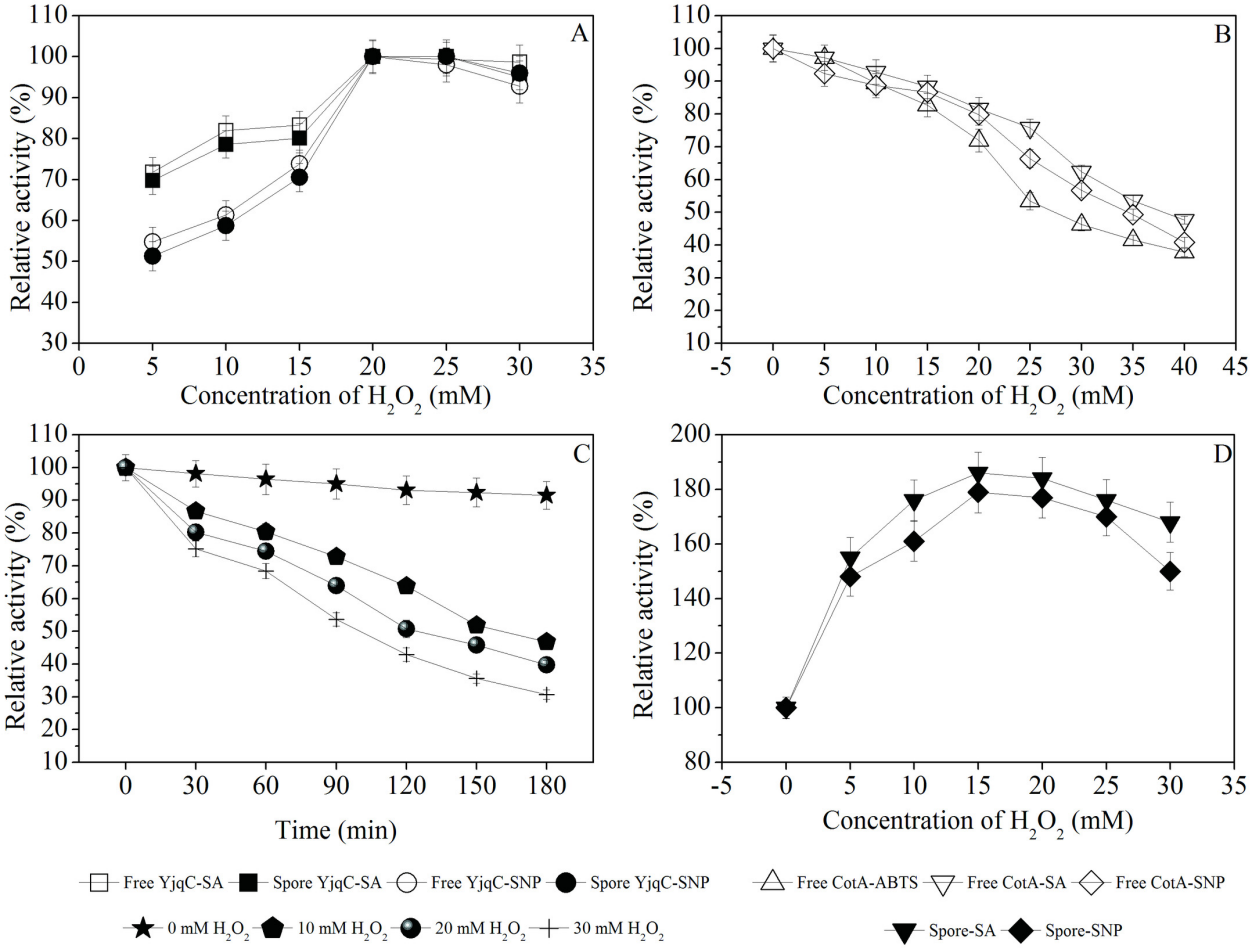
Effects of H_2_O_2_ concentrations on activity of YjqC and CotA from *B*. *altitudinis* hb4. (A) Variable H_2_O_2_ effects on peroxidase activity from the free and spore YjqC. (B) Inhibition by H_2_O_2_ of CotA activity toward ABTS, SA and SNP. (C) Stability of CotA incubated with different concentrations of H_2_O_2_ for 0–180 min. (D) Effects of H_2_O_2_ concentrations on enzymatic activity of spore enzymes toward SA and SNP.

As shown in Fig 3C and 3D, the free YjqC could catalyze the oxidation of SA and SNP under a pH range of 5.0–9.0 and a temperature range of 40–80°C. The optimal pH values for the free YjqC toward SA and SNP were 8.0 and 7.5, respectively. The optimal temperatures for the free YjqC toward SA and SNP were 60 and 70°C, respectively. As shown in Fig 5A, the free YjqC exhibited increased peroxidase activity toward SA and SNP (1 mM of final concentration) as the H_2_O_2_ concentration increased until 20 mM. A certain amount of H_2_O_2_ is the co-substrate used for driving peroxidase activity for the free YjqC [26]. In the presence of 20 mM H_2_O_2_, free YjqC exhibited increased peroxidase activity toward SA (0.135–0.81 mM) and SNP (0.016–0.096 mM) (Fig 4B and 4C). The kinetic parameters of YjqC toward SA and SNP followed Michaelis–Menten kinetics. As shown in Table 4, the *K*_M_ values suggested that YjqC had a higher affinity for SA than SNP. The *k*_cat_/*K*_M_ values also suggested that the free YjqC exhibited a higher catalytic efficiency toward SA than SNP. Compared to CotA, the YjqC has a higher *K*_M_ value toward SA than SNP. Their *k*_cat_/*K*_M_ values suggest that the catalytic efficiencies of the YjqC toward SA and SNP are inferior to the efficiencies of CotA.

Based on above analyses, we confirmed that YjqC has peroxidase activity toward SA and SNP. According to what we have learned, this is the first report about the peroxidase activity of *Bacillus* YjqC. Catalases may have peroxidase activity when they encounter specific substrates [15, 17]. In this study, SA and SNP evoked peroxidase activity of *B*. *altitudinis* hb4 YjqC. Generally speaking, enzymes with low *K*_M_ are more efficient than enzymes with high *K*_M_ with low levels of substrates. However, enzymes with low *K*_M_ may more vulnerable to inactivation by high levels of substrate [17]. The YjqC and CotA of *B*. *altitudinis* hb4 showed different affinity and catalytic efficiency for SA and SNP. Therefore, they would be complementary for the catalytic oxidation of SA and SNP distributed in a complex system.

### Characterization of Spore YjqC and CotA

Compared to the free YjqC, the spore YjqC exhibited an increased *K*_M_ and a decreased *V*_max_ (Table 4). The spore YjqC exhibited optimal catalase activity at pH 8.0 and 45°C (Fig 3A and 3B). In contrast, spore catalase of *B*. *pumilus* strains SAFR-032 and ATCC 7061 showed optimal activity at 70 and 37°C, respectively [13]. The spore YjqC also exhibited peroxidase activity toward SA and SNP. The spore YjqC exhibited optimal peroxidase activity toward SA at pH 8.0 and 65°C. The optimal conditions for the spore YjqC toward SNP were pH 8.0 and 70°C. Compared to the free YjqC, the spore YjqC had a lower catalytic efficiency toward SA and SNP. Similar to the free YjqC, the spore YjqC exhibited a slightly higher affinity and catalytic efficiency for SA than SNP.

Compared to free CotA, the optimal conditions for the spore CotA toward ABTS shifted to pH 4.5 and 85°C. The optimal pH of the spore CotA toward SA and SNP shifted to 9.0 and 7.0, respectively (Fig 2C). The optimal temperature for the spore CotA toward SA and SNP increased from 65 to 75°C, respectively (Fig 2D). Compared to the free CotA, the spore CotA showed an increased *K*_M_ and a decreased *V*_max_ toward ABTS, SA and SNP (Table 4). In a comparative analysis of *V*_max_/*K*_M_ values, the spore CotA of *B*. *altitudinis* hb4 showed a much higher catalytic efficiency toward ABTS than SA and SNP. It exhibited a higher affinity for SNP than SA. Compared with the CotA laccase on *B*. *subtilis* spores, *B*. *altitudinis* hb4 spore CotA had a much higher *K*_M_ value and a lower *V*_max_/*K*_M_ toward ABTS (Table D in S2 File). Unfortunately, there are few reports about the use of the spore CotA or YjqC for catalyzing the oxidation of SA and SNP for comparison.

The increased *K*_M_ value and decreased catalytic efficiency of the spore YjqC or CotA can largely be attributed to spore coat being an obstacle for substrate diffusion or product emission. This phenomemon has also appeared for the immobilization of enzymes in spores [21]. Althoug there were disadvantages of spore YjqC or CotA, they had much higher stability than free enzymes under harsh conditions such as alkali pH, heat and high concentrations of chemicals [36]. The free CotA had only approximately 45% of residual activity after 12 h of incubation under the optimal pH and temperature. In constrast, the spore CotA exhibited higher stability under the optimal conditions. The spore CotA retained approximately 70% of its residual activity toward ABTS after an incubation of 12 h (Fig 2E). Similarly, the spore CotA also had better stability than free CotA for catalyzing the oxidation of SA and SNP (Fig 2F). The spore YjqC also exhibited a higher stability than free YjqC. The residual activity of the spore YjqC was 40% higher than the residual activity for free YjqC after an incubation of 12 h under optimal conditions (Fig 3E). The spore also elevated the stability of the YjqC-catalyzed oxidation of SA and SNP under optimal conditions (Fig 3F).

### H_2_O_2_ Stability of CotA

As shown in Fig 5B, activity of CotA was inhibited by H_2_O_2_. The IC50 values of H_2_O_2_ against activity of *B*. *altitudinis* hb4 CotA were calculated to be approximately 27.5, 38 and 35 mM for ABTS, SA and SNP, respectively. CotA of *B*. *altitudinis* hb4 had a much higher H_2_O_2_ tolerance than laccases from *Trametes versicolor* and *Trametes trogii* (Table 5). The activity of the *Trametes trogii* laccase was inhibited by 50% at only 165 μM H_2_O_2_. Compared with the versatile ligninolytic peroxidase of *Pleurotus eryngii*, *B*. *altitudinis* hb4 CotA had a much longer half-life for catalytic activity (Table 5).

**Table 5:** Effects of H_2_O_2_ on activity of laccase and peroxidase toward ABTS.

Enzyme	Strain	IC50 of H_2_O_2_ against activity	Catalytic stability halflife (min)	Reference
Laccase	*Trametes versicolor*	10 mM	ND^a^	[6, 8, 45]
Laccase	*Trametes trogii*	165 μM	ND	[7]
VP^b^	*Pleurotus eryngii*	ND	4.1^c^	[3]
CotA	*B*. *altitudinis* hb4	27.5 mM	100–160^d^	This study

^*a*^ No data provided

^*b*^ Versatile ligninolytic peroxidase

^*c*^ Incubation of enzyme with 1 mM H_2_O_2_

^*d*^ Incubation of enzyme with 10, 20 and 30 mM H_2_O_2_

As shown in Fig 5C, the half-life of CotA decreased with increasing H_2_O_2_ concentrations. The past study demonstrated that the impaired activity of laccase was attributed to inhibition rather than denaturation by H_2_O_2_ [7]. It was initially concluded that the H_2_O_2_ stability of *B*. *altitudinis* hb4 CotA was much higher than the stability of laccase and peroxidase from fungi. The striking H_2_O_2_ stability of *B*. *altitudinis* hb4 CotA might be the common properties of CotA laccases from the *B*. *pumilus* group. This similarity would be benefical for developing biotechnological applications for CotA.

A certain concentration of H_2_O_2_ was indispensable for the YjqC-catalyzed oxidation of SA and SNP. However, high concentrations of H_2_O_2_ had an inhibitory effect on the laccase activity of CotA [6]. At 20 mM H_2_O_2_, the free CotA showed approximately 82 and 80% of relative activity toward SA and SNP, respectively (Fig 5B). Previous studies have demonstrated that spore maturation leads to a minor improvement in the enzymatic properties of CotA, such as resistance to proteinase digestion [22]. Therefore, spores might relieve inhibition of H_2_O_2_ to CotA. The effects of H_2_O_2_ on the spore-catalyzed oxidation of SA and SNP were also determined and compared (Fig 5D). Without the effects of H_2_O_2_, the activity of spores oxidizing SA and SNP was denoted as 100%. The spores showed increased catalytic activity toward SA and SNP with increases in H_2_O_2_. At 15 mM H_2_O_2_, the spores exhibited maximum activity toward SA (186%) and SNP (179%), respectively. Therefore, the combination of *B*. *altitudinis* hb4 spores and 15 mM H_2_O_2_ was powerful for the oxidation of SA and SNP.

### Spore-Catalyzed Degradation of SA and SNP in RSM

**Fig 6:**
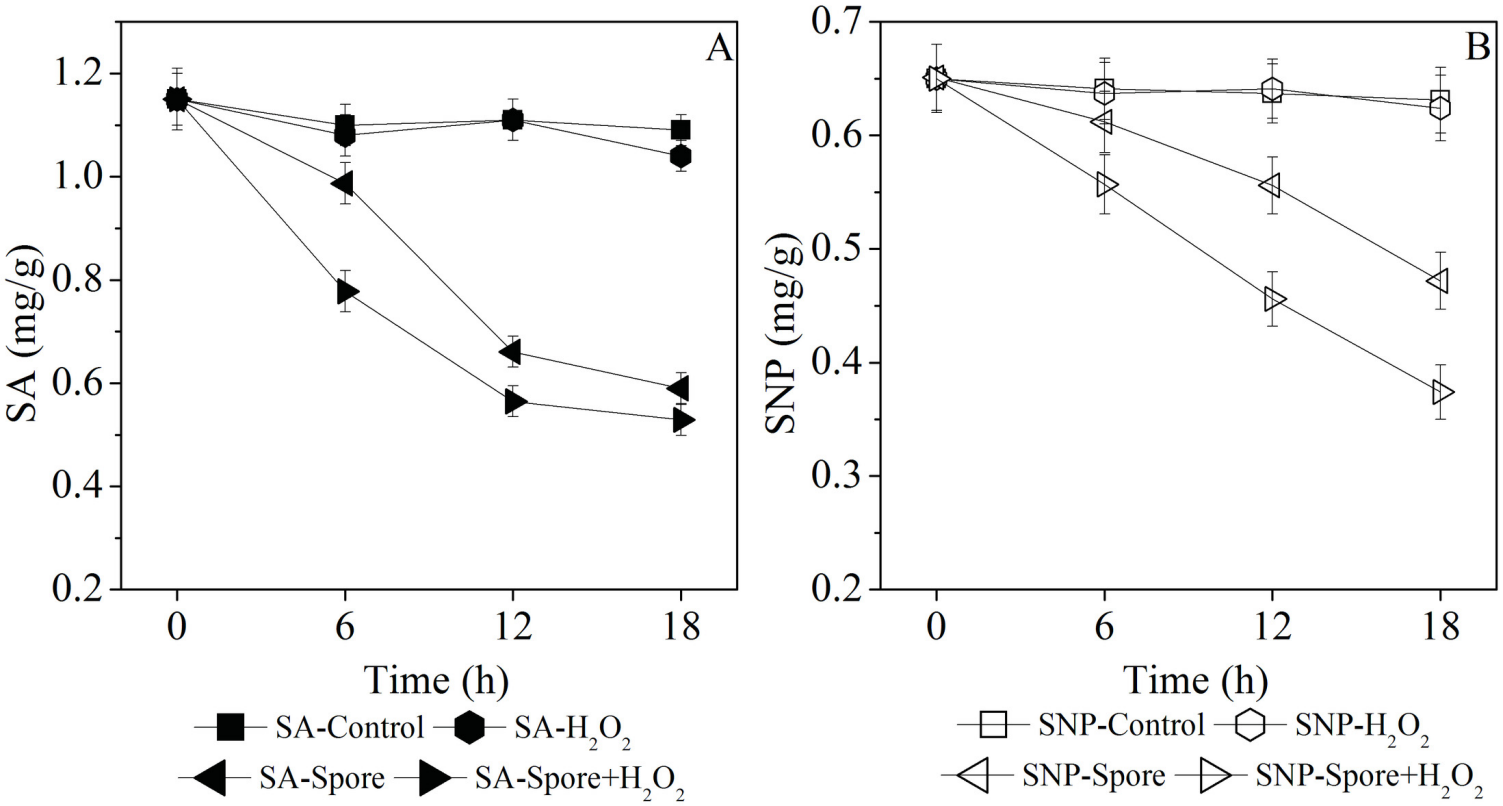
(A) Contents of SA extracted from RSM treated with 15 mM H_2_O_2_ and/or spores for 18 h. (B) Contents of SNP extracted from RSM treated with 15 mM H_2_O_2_ and/or spores for 18 h.

As shown in Fig 6A and 6B, there was less SA and SNP extracted from the RSM treated with the spore suspension. Approximately 1.15 mg/g of SA was extracted from the RSM with no treatment. In contrast, only 0.59 mg/g of SA was extracted from the RSM treated with the spore suspension for 18 h. The amount of SA extracted decreased to 0.53 mg/g when RSM was treated with the spore suspension plus 15 mM H_2_O_2_. The content of SA was not reduced by the sole use of 15 mM H_2_O_2_ for treating RSM. Similar changes were also observed in the amount of SNP extracted from RSM (Fig 6B). Without any treatment, approximately 0.65 mg/g of SNP was extracted from RSM. Following treatment of RSM with the spore suspension, the amount of SNP decreased to 0.47 mg/g. The amount of SNP further decreased to 0.37 mg/g with the addition of 15 mM H_2_O_2_ to the spore suspension. Compared with the controls, a similar amount of SNP was extracted from the RSM treated with H_2_O_2_. In conclusion, the SA and SNP content decreased by 46 and 25% after treating RSM with the spore suspension for 18 h. The SA and SNP content decreased approximately 51 and 41% after RSM was treated with the spore suspension plus 15 mM H_2_O_2_. These results demonstrated that the spores were efficient in the catalytic degradation of SA and SNP in RSM. Moreover, this spore-catalyzed degradation could be improved by adding H_2_O_2_. The spore-catalyzed degradation of SNP was increased by 64% with 15 mM H_2_O_2_. The elevated efficiency might have occurred due to the activation of peroxidase activity from the spore YjqC.

As mentioned previously, the spores could be regarded as an inherent multi-enzyme biocatalyst comprising YjqC and CotA. Numerous studies have demonstrated that a multi-enzyme biocatalyst is more promising for the degradation of phenolic compounds in biomass [46]. Moreover, the spores have a much higher H_2_O_2_ stability than free YjqC and CotA. The spores are also beneficial for enzyme purification and immobilization processes. The industrial-scale production of spores can also be more easily implemented. Thus, there is great potential for utilizing spores as biocatalyst in catalyzing the degradation of SA and SNP in RSM. Due to the relative ubiquity of YjqC and CotA in *Bacillus* species, the utilization of *B*. *altitudinis* hb4 spores for treating RSM could provide an ideal experience for other *Bacillus* strains, especially microbial feed additives such as *B*. *pumilus* and *B*. *subtilis*.

## Supporting Information

S1 File Supporting data for Figs A-D. Fig. A Neighbor-joining tree exhibiting the phylogenetic relationship of *B*. *altitudinis* SYBC hb4 CotA and YjqC to homologous proteins from other *Bacillus* strains. Fig. B (a) The standard curve exhibited a good linear relationship between peak area and concentration of standard SNP. (b) HPLC-DAD chromatograms of standard SNP. Fig. C (a) The standard curve exhibited a good linear relationship between peak area and concentration of standard SA. (b) HPLC-DAD chromatograms of standard SA. Fig. D HPLC-DAD chromatograms of a mixture of standard SA and standard SNP.
(DOC)

S2 File Supporting data for Tables A-D. Table A. Kinetic properties of purified laccase from *Bacillus* species toward ABTS. Table B. Kinetic properties of Mn catalase or peroxidase. Table C. Kinetic properties of purified laccase toward SA. Table D. Kinetic properties of spore laccase toward ABTS.
(DOC)
