# Supplementary material for: Hydrogen Peroxide-Resistant CotA and YjqC of Bacillus altitudinis Spores Are a Promising Biocatalyst for Catalyzing Reduction of Sinapic Acid and Sinapine in Rapeseed Meal
Source: PLoS One. 2016 Jun 30;11(6):e0158351. doi: 10.1371/journal.pone.0158351 (PMC4928806; doi:10.1371/journal.pone.0158351)
Supplement: S1 File — Fig. A Neighbor-joining tree exhibiting the phylogenetic relationship of B. altitudinis SYBC hb4 CotA and YjqC to homologous proteins from other Bacillus strains. Fig. B (a) The standard curve exhibited a good linear relationship between peak area and concentration of standard SNP. (b) HPLC-DAD chromatograms of standard SNP. Fig. C (a) The standard curve exhibited a good linear relationship between peak area and concentration of standard SA. (b) HPLC-DAD chromatograms of standard SA. Fig. D HPLC-DAD chromatograms of a mixture of standard SA and standard SNP. (DOC) [file pone.0158351.s001.doc]

*Bacillus altitudinis* manganese catalase (WP_012010687.1)

*Bacillus aerophilus* manganese catalase (WP_041507114.1)

*Bacillus pumilus* manganese catalase (WP_024718350.1)

***Bacillus altitudinis* SYBC hb4 YjqC (AHI58963.1)**

*Bacillus amyloliquefaciens* manganese catalase (WP_040238256.1)

*Bacillus subtilis* manganese catalase (KFF56732.1)

*Bacillus licheniformis* manganese catalase (WP_044789435.1)

*Bacillus amyloliquefaciens* copper oxidase (WP_045508081.1)

*Bacillus subtilis* spore coat protein A (WP 015482891.1)

*Bacillus licheniformis* copper oxidase (WP_011197606.1)

*Bacillus aerophilus* copper oxidase (WP 041506688.1)

*Bacillus pumilus* spore coat protein A (WP 025206837.1)

***Bacillus altitudinis* SYBC hb4 CotA (KU363621)**

*Bacillus altitudinis* spore coat protein A (WP_035703508.1)

64

60

100

100

100

100

89

100

95

78

0.05

**Fig. A Neighbor-joining tree exhibiting the phylogenetic relationship of *B. altitudinis* SYBC hb4 CotA and YjqC to homologous proteins from other *Bacillus* strains.**

(a)

(b)

**Fig. B (a) The standard curve exhibited a good linear relationship between peak area and concentration of standard SNP. (b) HPLC-DAD chromatograms of standard SNP.**

(a)

(b)

**Fig. C (a) The standard curve exhibited a good linear relationship between peak area and concentration of standard SA. (b) HPLC-DAD chromatograms of standard SA.**

**Fig. D HPLC-DAD chromatograms of a mixture of standard SA and standard SNP.**
