# Supplementary material for: Hydrogen Peroxide-Resistant CotA and YjqC of Bacillus altitudinis Spores Are a Promising Biocatalyst for Catalyzing Reduction of Sinapic Acid and Sinapine in Rapeseed Meal
Source: PLoS One. 2016 Jun 30;11(6):e0158351. doi: 10.1371/journal.pone.0158351 (PMC4928806; doi:10.1371/journal.pone.0158351)
Supplement: S2 File — Table A. Kinetic properties of purified laccase from Bacillus species toward ABTS. Table B. Kinetic properties of Mn catalase or peroxidase. Table C. Kinetic properties of purified laccase toward SA. Table D. Kinetic properties of spore laccase toward ABTS. (DOC) [file pone.0158351.s002.doc]

**Table A. Kinetic properties of purified laccase from *Bacillus* species toward ABTS.**

| **Strain** | **Enzyme** | ***K*_M_ (μM)** | ***k*_cat_/*K*_M_ (μM^-1^s^-1^)** | **Opt pH** | **Opt Tem (°C)** | **Reference** |
| --- | --- | --- | --- | --- | --- | --- |
| *B. subtilis* | rCotA | 124 | 2.6 | ND*^a^* | ND |  |
| *B. subtilis* WD23 | rCotA | 162 | 0.09 | 4.5 | 80 |  |
| *B. licheniformis* DSM 13 | rCotA | 6.5 | 12.8 | 4.2 | 85 |  |
| *B. coagulans* DSM1 | rLMCO | 31 | 2.22 | 4 | ND |  |
| *B. clausii* DSM8716 | rLMCO | 132 | 0.68 | 4 | ND |  |
| *B. pumilus* DSM 27 | rCotA | 80 | 3.64 | 4 | 70 |  |
| *B. vallismortis* fmb-103 | Laccase | 22.7 | ND | 4.8 | 82 |  |
| *B. subtilis* 168 | rCotA | 106 | 0.498 | 4.0 | 40 |  |

*^a^* No data provided

**Table B. Kinetic properties of Mn catalase or peroxidase.**

| **Strain** | **Enzyme** | **Substrate** | ***K*_M_ (mM)** | ***k*_cat_/*K*_M_ (μM^-1^s^-1^)** | **Assay conditions** | | **Reference** |
| --- | --- | --- | --- | --- | --- | --- | --- |
|  |  |  |  |  | **pH** | **Temperature (°C)** |  |
| *B. subtilis* 168 | DyP | ABTS | 166 | 0.07 | 4.0 | 25 |  |
| *P. calidifontis* | Mn-CAT | H_2_O_2_ | 170 | 0.17 | 7.0 | 25 |  |
| *L. plantarum* | Mn-CAT | H_2_O_2_ | 350 | 0.094 | ND*^a^* | ND |  |
| *T. thermophilum* | Mn-CAT | H_2_O_2_ | 83 | 3.1 | ND | ND |  |
| *T. album* | Mn-CAT | H_2_O_2_ | 15 | 0.41 | 8.0-9.0 | 25-35 |  |
| *T. fusca* | TfuCat | H_2_O_2_ | 49 | 10.26 | 9.0 | 74 |  |
| *T. fusca* | TfuCat *^b^* | catechol +H_2_O_2_ | ND | ND | 8.0-10.0 | 74 |  |

*^a^* ND, no data provided

*^b^* TfuCat exhibited peroxidase activity toward equimolar amounts of catechol and H_2_O_2_ (50 mM) with *k*_cat_ value of 975 s^−1^

**Table C. Kinetic properties of purified laccase toward** SA.

| **Strain** | **Enzyme** | ***K*_M_ (μM)** | ***k*_cat_/*K*_M_ (μM^-1^s^-1^)** | **Assay conditions** | | **Reference** |
| --- | --- | --- | --- | --- | --- | --- |
|  |  |  |  | **pH** | **Temperature (°C)** |  |
| *B. subtilis* 168 | rCotA | 480 | 0.082 | 7.0 | 40 |  |
| *C. hirsutus* | Lac | 11 | ND | 4.0 | 70 |  |
| *T*. *hirsuta* | Lac | 24 | 24.2 | ND*^a^* | ND |  |
| *T*. *ochracea* | Lac | 11 | 15.4 | ND | ND |  |
| *C. maxima* | Lac | 24 | 13.7 | ND | ND |  |
| *C. fulvocinerea* | Lac | 21 | 6.67 | ND | ND |  |

*^a^* ND, no data provided

**Table D. Kinetic properties of spore laccase toward** ABTS.

| **Strain** | **Enzyme** | ***K*_M_ (μM)** | ***V*_max_** | ***V*_max_/ *K*_M_** | **Opt pH** | **Opt Tem (°C)** | **Reference** |
| --- | --- | --- | --- | --- | --- | --- | --- |
| *Armillaria* sp. F022 | sLac | 325 | 95*^a^* | 0.29*^a^* | 4.0 | 37 |  |
| *B. subtilis* 1S101 | wtCotA displayed on spores | 58.8 | 33.3*^b^* | 0.56*^b^* | 4.0 | 25 |  |
| *B. subtilis* 1S101 | wtCotA displayed on spores | 57.8 | 26.7*^b^* | 0.46*^b^* | 4.0 | ND*^c^* |  |

*^a^* Unit of *Vmax* and *Vmax*/*K_M_* was U mg^-1^ spores and l mg spores^−1^ min^−1^

*^b^* Unit of *Vmax* and *Vmax*/*K_M_* was U OD_580_ of spores^-1^ and min^-1^ OD_580_ of spores^-1^

*^c^* ND, no data provided

**References:**

1. Koschorreck K, Richter S, Ene A, Roduner E, Schmid R, Urlacher V. Cloning and characterization of a new laccase from *Bacillus licheniformis* catalyzing dimerization of phenolic acids. Appl Microbiol Biotechnol. 2008;79(2):217-24. doi: 10.1007/s00253-008-1417-2.

2. Fan LL, Zhao M, Wang Y. Expression of CotA laccase in Pichia pastoris and its electrocatalytic sensing application for hydrogen peroxide. Appl Microbiol Biotechnol. 2015;99(22):9483-93. PubMed PMID: WOS:000363951800014.

3. Ihssen J, Reiss R, Luchsinger R, Thony-Meyer L, Richter M. Biochemical properties and yields of diverse bacterial laccase-like multicopper oxidases expressed in *Escherichia coli*. Sci Rep-Uk. 2015;5:10465. PubMed PMID: WOS:000356149100001.

4. Reiss R, Ihssen J, Thony-Meyer L. Bacillus pumilus laccase: a heat stable enzyme with a wide substrate spectrum. Bmc Biotechnology. 2011;11. PubMed PMID: WOS:000287475500001.

5. Zhang C, Zhang S, Diao H, Zhao H, Zhu X, Lu F, et al. Purification and characterization of a temperature- and pH-stable laccase from the spores of *Bacillus vallismortis* fmb-103 and its application in the degradation of malachite green. J Agric Food Chem. 2013;61(23):5468-73. doi: 10.1021/jf4010498.

6. Xie T, Liu Z, Liu Q, Wang G. Structural insight into the oxidation of sinapic acid by CotA laccase. J Struct Biol. 2015;190(2):155-61.

7. Santos A, Mendes S, Brissos V, Martins LO. New dye-decolorizing peroxidases from *Bacillus subtilis* and *Pseudomonas putida* MET94: towards biotechnological applications. Appl Microbiol Biotechnol. 2014;98(5):2053-65. PubMed PMID: WOS:000332107700012.

8. Amo T, Atomi H, Imanaka T. Unique presence of a manganese catalase in a *Hyperthermophilic Archaeon*, *Pyrobaculum calidifontis* VA1. J Bacteriol. 2002;184(12):3305-12.

9. Lonear N, Fraaije M. Not so monofunctional-a case of thermostable *Thermobifida fusca* catalase with peroxidase activity. Appl Microbiol Biotechnol. 2015;99(5):2225-32. doi: 10.1007/s00253-014-6060-5.

10. Shin KS, Kim CJ. Properties of laccase purified from nitrogen limited culture of white-rot fungus *Coriolus hirsutus*. Biotechnol Tech. 1998;12(2):101-4. PubMed PMID: WOS:000072176100004.

11. Shleev SV, Morozova O, Nikitina O, Gorshina ES, Rusinova T, Serezhenkov VA, et al. Comparison of physico-chemical characteristics of four laccases from different *basidiomycetes*. Biochimie. 2004;86:693-703. PubMed PMID: WOS:000225621600013.

12. Hadibarata T, Yusoff ARM, Aris A, Salmiati, Hidayat T, Kristanti RA. Decolorization of azo, triphenylmethane and anthraquinone dyes by laccase of a newly isolated *Armillaria* sp F022. Water Air Soil Poll. 2012;223(3):1045-54. PubMed PMID: WOS:000303385500008.

13. Jia H, Lee FS, Farinas ET. *Bacillus subtilis* spore display of laccase for evolution under extreme conditions of high concentrations of organic solvent. Acs Comb Sci. 2014;16(12):665-9. PubMed PMID: WOS:000346114600002.

14. Gupta N, Farinas ET. Directed evolution of CotA laccase for increased substrate specificity using *Bacillus subtilis* spores. Protein Engineering Design and Selection. 2010;23(8):679-82.
